# Spatio-Temporal Characteristics of Trade-Offs and Synergies in Ecosystem Services at Watershed and Landscape Scales: A Case Analysis of the Yellow River Basin (Henan Section)

**DOI:** 10.3390/ijerph192315772

**Published:** 2022-11-27

**Authors:** Haipeng Niu, Mengmeng Liu, Dongyang Xiao, Xiaoming Zhao, Ran An, Liangxin Fan

**Affiliations:** 1 School of Surveying and Land Information Engineering, Henan Polytechnic University, Jiaozuo 454000, China; 2 Research Centre of Arable Land Protection and Urban-Rural High-Quality Development of Yellow River Basin, Henan Polytechnic University, Jiaozuo 454000, China; 3 School of Resources & Environment, Henan Polytechnic University, Jiaozuo 454000, China

**Keywords:** Yellow River Basin, ecosystem services, InVEST model, spatio-temporal assessment, trade-offs and synergies, multi-levels

## Abstract

The changes and interrelationships of ecosystem services at different global and regional scales have been actively investigated. Clarifying the trade-offs and synergies between ecosystem services from a multi-scale scientific perspective is vital to improve the coordinated and sustainable development of the watershed and ecological protection. As an important ecological barrier region of the Yellow River Basin, the Henan section provides a variety of important ecosystem services. This study analyzes the characteristics of land use changes in the Yellow River Basin (Henan section) from 1990 to 2020. Based on the InVEST model, four ecosystem services—water production, soil conservation, carbon storage and food supply have been evaluated. The Spearman correlation coefficient was used to further reveal the spatial and temporal characteristics of the trade-offs and synergies at different levels of each service. The results showed that: (1) From 1990 to 2020, the basin was dominated by farmland conservation. The construction land area mainly exhibited an inflow behavior, while other land use types were mainly related to outflow. (2) From 1990 to 2020, the water yield, soil conservation and carbon storage first increased and then decreased, while food supply gradually increased. The spatial distribution of these ecosystem services was lower in the southwest and slightly higher in the northeast and farmland had the highest capacity of water production and food supply, while woodland had the highest capacity for soil conservation and carbon storage. (3) The Spearman rank correlation coefficient indicated that the trade-offs for the ecosystem services in the Yellow River Basin (Henan section) dominated before 2000, and the synergies gradually strengthened after 2000. (4) There were clear spatial heterogeneities in the ecosystem services of the basin; for instance, the functions in the middle and lower reaches of the Yellow River Basin (Henan section) were mainly trade-offs, while the higher elevations in the middle reaches exhibited synergistic relationships. This study aims to clarify the trade-offs and synergies between ecosystem services at the different levels. Based on our findings, countermeasures and suggestions for ecological protection and management are proposed to promote the coordinated development of social economy and ecological protection.

## 1. Introduction

As natural resources and assets, ecosystems play an indispensable role in the development and survival of human beings [1]. Ecosystem services refer to the supply of various products and services required to meet the requirements of and maintain human life in natural ecosystems and ecological processes, which are closely related to human well-being [2,3]. The United Nations’ Millennium Ecosystem Assessment Plan divides the field into four categories: supply, regulation, culture and support services [4]. Long-term acceleration of human resources development and land use to meet the growing population demand, exceeding the carrying capacity of the ecosystem, leads to ecological degradation and environmental problems such as habitat fragmentation, soil erosion and water pollution [5,6]. At present, the global action to prevent ecosystem degradation is emerging, which has stimulated a lot of exploratory research, and the study of ecological service function assessment and trade-off synergistic relationships has also become the research focus. It is vital to clarify the interactions among ecosystem services to improve regional coordination and sustainable development and achieve a “win—win” outcome with respect to ecological conservation [7,8].

At a certain scale, ecosystem services are not completely unrelated, but affect each other in a variety of complicated ways [9]. The pros and cons of trade-offs and mutually reinforcing synergies are typical manifestations of this effect [10,11]. When an increase or decrease in one ecosystem service is accompanied by a decrease or increase in another ecosystem service, the relationship is referred to as a trade-off. When two ecosystem services increase or decrease simultaneously, it is referred to as synergistic [12]. In the presence of such complex relationships, the changes of ecosystem services are closely related. The drastic changes of land use over the past 50 years have influenced the ecosystem pattern and prompted changes in ecosystem services and their relationships. Studying the spatial and temporal changes and interrelationships of ecosystem services and exploring their regional differences and level effects can provide a basic reference for regional land use decision-making and ecosystem service management [13].

The present studies mainly used the theories of geography and ecology to perform qualitative analyses of ecosystem services, but quantitative studies on the service benefits were few [14]. Researchers typically used statistical descriptions [15], simulation of scenarios [16] and quantitative modelling [17] to conduct assessments at different spatial scales such as regions, watersheds, mountainous areas and ecological function areas, or different systems such as forests, farmland and grassland, then further analyzed the coupling relationship between the different functions. Marques et al. [18] used the water yield (*WY*) of the InVEST model to perform a functional assessment of the Francoli Basin in Spain and further explored the response of water production to climate change. Goldman et al. [19] used the water yield and nutrient transport module in the InVEST model to evaluate the ecosystem service function in Colombia and analyzed the correlations in order to formulate a more scientific investment plan. Li et al. [20] used the modules of water yield, soil conservation (*SC*) and water quality purification in the InVEST model to study the impact of land use change on ecosystem services in the Miyun Reservoir Basin. Li et al. [21] explored the changes of land use and ecosystem services in the mainstream and tributaries of the Weihe River Basin for assessment and classification with the help of the InVEST model; they also used a correlation analysis to analyze the trade-off and synergistic relationships between the various service functions. Yang et al. [22] evaluated five ecosystem services, namely water yield, carbon storage (*CS*), soil conservation, NPP and habitat quality, in the Yellow River Basin via the InVEST and CASA models and analyzed the trade-off for the relationships among the various ecosystem services. Most of the above scholars’ research on the ecosystem service assessment and the trade-offs and synergies were based on quantitative analysis of large and medium scale watersheds or administrative boundaries, while research tended to lack in the trade-offs and synergies of ecosystem services at the scale of small basins and multi-levels service functions. In addition, there was little research on the dynamic trends and driving mechanisms of the service changes (natural and anthropogenic inputs and impacts) under long-term sequences. Therefore, research on the trade-offs and synergies of ecosystem services needs to eliminate the previous boundary selection and single-scale model defects and carry out multi-scale and multi-type service correlation research to clarify the trade-offs and synergies and spatial differences among the multi-type services, so as to facilitate the effective implementation of ecosystem management.

The Yellow River Basin is an important ecological barrier area and economic zone in China, performing vital ecological functions and occupying an indispensable position in maintaining environmental security and social and economic development [23]. The “Ecological protection and high-quality development of the Yellow River Basin” in the “14th Five-Year Plan” has been identified as a major regional development strategy in China, and strategic research results have emerged constantly. Additionally, progress has been made in various fields such as ecological protection [24,25], the industrial economy [26,27] and ecological efficiency [28,29]. The ecosystems of the upper, middle and lower reaches of the Yellow River Basin largely differ. The Yellow River Basin (Henan section) covers both the middle and lower reaches, among which soil erosion in the middle reaches is serious and the ecological flow in the lower reaches is lower [30]. As a small watershed, the Yellow River Basin (Henan section) provides multiple types of ecosystem services and is a targeted study area. Most studies have been based on the whole basin or administrative boundaries, but targeted and in-depth research on the ecological functions and relationships of small basins at the spatial level is lacking.

In summary, previous studies on ecosystem services have focused on arid/semi-arid areas, mostly at large and medium scales such as the Yellow River Basin and the Weihe River Basin. There were few studies on the trade-offs and synergies between services in different land use types and the sequence was short. Therefore, this article eliminated previous studies based on the Yellow River Basin or administrative boundary, and selected key regions in the Yellow River Basin based on the watershed boundary to carry out the trade-off and synergy of long-term (1990–2020) and multi-scale (watershed and landscape) ecosystem services research for the semi-humid temperate climate zones, which provides a scientific and practical reference for the study of ecosystem services and the protection of mountains, forests, fields, lakes, grasses and sand in the semi-humid climate zones.

## 2. Materials and Methods

### 2.1. Overview of Study Area

**Figure 1:**
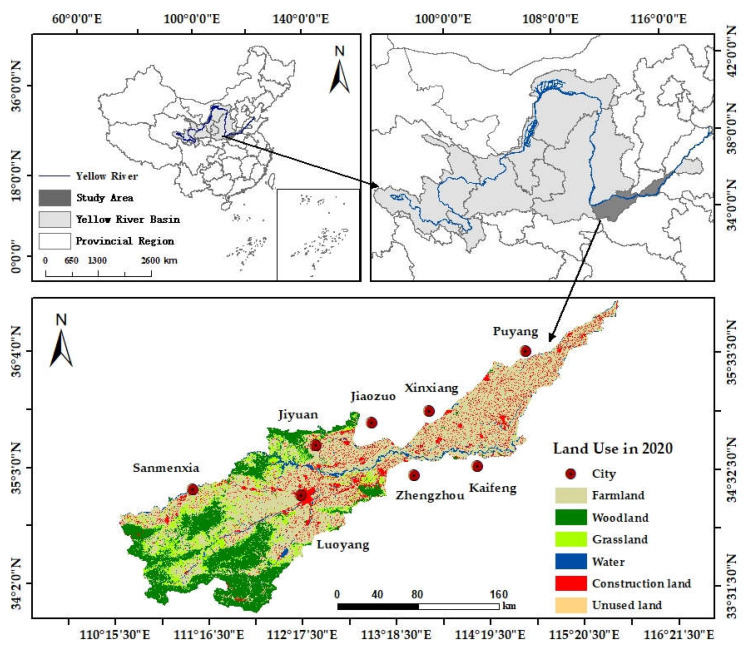
Schematic of the Yellow River Basin (Henan section). Notes: The data of land use cover in 2020 used in the figure was from Landsat 8 OLI remote sensing image data with a spatial resolution of 30 m.

The source of the Yellow River is in the Bayan Har Mountain range on the Qinghai-Tibet Plateau with the river eventually reaching Kenli Country, Shandong Province to join the Bohai Sea. The main section of the river is 5464 km in length, 1900 km from east to west and 1100 km from north to south. The terrain is high in the east and low in the west, with a drop of 4480 m. The Yellow River Basin flows through nine provinces and regions from west to east, and in the Henan section, which covers the middle and lower reaches of the basin, the total length is 711 km and the total area is 36,500 km^2^. The river flows into Henan from Tongguan County, starting from Lingbao City in the west of the province, and joins the lower reaches of the river after Taohua Valley at Zhengzhou, then flowing through Taiqian County into Shandong [31]. Overall, the river flows from west to east through eight major cities and twenty six counties (cities and districts) (Figure 1).

The Yellow River Basin (Henan section) is located in the transition zone known in China as the second to third ladders. The region has a semi-arid and semi-humid climate within a warm temperate zone; specifically, the region has a continental monsoon climate within the transition from the northern subtropical zone to the warm temperate zone. In addition, the basin transitions from a mountainous to a plain climate from west to east, and the overall terrain is high in the west and low in the east [32]. The annual average precipitation is 440–696 mm, the annual average temperature is 12–15° and the annual average evapotranspiration is 617–1090 mm. The rainfall is unevenly distributed in season and space, with the trend of more rain in the south and less in the north. There is low precipitation and high evaporation in spring, abundant rainfall and high air humidity in summer and sufficient sunshine and little rainfall in autumn. The weather is dry and cold in winter with little rain and snow. The period from June to September with abundant rain, elevated temperatures and extended daylight in the basin is beneficial to the growth of crops. In recent years, the development of tourism and the region economy has facilitated population increase in the river basin. According to the Statistical Yearbook 2021 of Henan Province, the permanent residential population of the Yellow River Basin (Henan section) was about 46.31 million in 2020, of which 29.26 million were the permanent urban residents, with an urbanization rate of 63.18%. The GDP of the region in 2020 was 3074.568 billion RMB, and the per capita GDP was 64,565.89 RMB per person.

### 2.2. Sources of Data

The main data used in this study included the land classification, meteorological date (such as rainfall and evapotranspiration), elevation, soil and other data (Table 1). The land use data for four periods from 1990 to 2020 were based on the cloud-free remote sensing image data from June to September, provided by the Chinese Academy of Sciences (https://www.resdc.cn/ (accessed on 20 July 2021)), with a spatial resolution of 30 m, among which the Landsat-TM/ETM images were used in 1990, 2000 and 2010, and Landsat 8 OLI images were used in 2020. The Kappa coefficient was generally of high accuracy (88.95%). The DEM data were obtained from the geospatial data cloud platform (https://www.gscloud.cn/ (accessed on 14 September 2021)), and operations such as filling depressions were performed. The meteorological data, which came from stations located near the Yellow River Basin (Henan section), were supplied by the China Meteorological Data Service Network (http://data.cma.cn (accessed on 14 September 2021)). The soil data were collected from the China Soil Database and Harmonized World Soil Database (1.2).

### 2.3. Study Methods

#### 2.3.1. Land Use Transfer Matrix

The transition matrix can map the structure of land classes at the beginning and the end of the research period, and also reflect the transfer and change among the various land types during the period in order to catalogue the transfer and source of the land types in the research period [33]. The expression is as follows:

(1) $$S_{ij}=\left|\begin{array}{cccc} S_{11} & S_{12} & \cdots & S_{1n}\\ S_{21} & S_{22} & \cdots & S_{2n}\\ \vdots & \vdots & \vdots & \vdots \\ S_{n1} & S_{n2} & \cdots & S_{nn} \end{array}\right|$$

where *S_jj_* represents the area of land use type *i* at the beginning of the study period and being transformed into land use type *j* at the end of the study period, and *n* indicates the number of land classes. The sum of each row represents the area of such land at the beginning of the study period, and the value of each row represents the direction and quantity of land transfer. The sum of each column represents the area of this type of land at the end of the study period, and the value of each column represents the direction and quantity of this type of land conversion.

#### 2.3.2. InVEST Model

(1) Water Yield. The calculation of water yield is based on the water balance equation for the grid unit, which is equal to the grid unit of rainfall minus evaporation. Meteorological factors, land use types and soil characteristics have an impact on the balance between rainfall and evaporation [34]. The basic principle of the module calculation process is as follows:

(2) $$Y_{x,j}=\left(1-\frac{AET_{x,j}}{P_{x}}\right)\times P_{x}$$

(3) $$\frac{AET_{x,j}}{P_{x}}=\frac{1+W_{x}\times R_{x,j}}{1+W_{x}\times R_{x,j}+\frac{1}{R_{x,j}}}$$

(4) $$W_{x}=Z\times \frac{AWC_{x}}{P_{x}}+1.25$$

(5) $$R_{x,j}=\frac{K_{x,j}\times ET_{0x}}{P_{x}}$$

(6) $$AWC_{x}=Min(MSD_{x},RD_{x})\times PAWC_{x}$$

In the expression, *Y_x,j_* represents the annual water yield (mm) of grid *x* on type *j* land; *AET_x,j_* denotes the average annual evapotranspiration (mm) of raster *x* on type *j* land; *P_x_* is the average annual rainfall (mm) of grid *x*; *AET_x,j_*/*P_x_* is based on the Budyko curve proposed by Fu [35] and Zhang [36]; *R_x,j_* represents the aridity index of grid *x* on type *j* land; *W_x_* is a dimensionless parameter used to describe climate and soil properties [37]; *Z* is a seasonal parameter for rainfall distribution and depth; *AWC_x_* represents the available water content of vegetation (mm) on grid *x*; *K_x,j_* represents the evapotranspiration coefficient of vegetation on raster *x* of type *j* land [38]; *ET*_0*x*_ represents the potential evapotranspiration (mm) on grid *x* [39]; *MSD_x_* and *RD_x_* represent the maximum soil depth and root depth, respectively.

(2) Soil Conservation. The difference between possible soil loss (*RKLS*) under bare land and real soil loss (*USLE*) under vegetation cover or engineering measures is assumed to represent soil conservation [40]. The basic principle of the calculation process for the module is as follows:

(7) RKLS=R×K×LS

(8) USLE=R×K×LS×P×C

(9) SD=RKLS−USLE

In the expression, *RKLS* represents the possible soil loss (t/(ha·a)); *USLE* represents the real soil loss (t/(ha·a)); *SD* denotes soil conservation (t/(ha·a)); *R* stands for the rainfall erosion factor (MJ·mm/(ha·h·a)); *K* is the soil erosion factor (t·ha·h/(ha·MJ·mm)); *LS* denotes the dimensionless slope length gradient factor; *P* is whether to take engineering measures (dimensionless); *C* is whether vegetation cover is adopted (dimensionless).

(3) Carbon Storage. The carbon storage is calculated as the total reserves including the aboveground carbon, underground carbon, soil carbon and decayed organic carbon in various land use types in the region [41]. The basic principle of module calculation is as follows:

(10) $$C_{tot}=C_{above}+C_{below}+C_{soil}+C_{dead}$$

In the expression, *C_tot_* represents the total carbon storage (t/ha); *C_above_* means the amount of carbon stored above the ground; *C_below_* indicates the amount of carbon stored below the ground; *C_soil_* represents the amount of carbon stored in the soil; *C_dead_* indicates the amount of carbon stored by organic matter in the litter.

#### 2.3.3. Food Supply

Food supply (*FS*) is one of the important ecological service functions of an agricultural ecosystem. As Henan is a major grain-producing province in China, the evaluation of the food supply function in the Yellow River Basin (Henan section) is of great significance. According to the existing land classification, vegetables, grains and oil-bearing seeds are classified as food supply from farmland, and meat and milk are classified as food supply from grassland. The supply of agricultural and pastoral products from two land types per unit area is calculated, respectively. The output of vegetables, grains, oil-bearing seeds, meat and milk are assigned to the grids of two land types by the *NDVI* value, and the supply data of agricultural and pastoral products are mapped spatially to evaluate their food supply capacity. The basic principle of the calculation is as follows:

(11) $$FS_{i}=\frac{NDVI_{i}}{NDVI_{sum}}\times G_{sum}$$

In the expression, *FS_i_* represents the total output of various grains assigned to the *i* grid; *G_sum_* represents the total amount of the various agricultural and pastoral products provided by farmland and grassland; *NDVI_i_* denotes the vegetation index of the *i* grid; *NDVI_sum_* is the sum of vegetation indices of the farmland and grassland in the study area.

#### 2.3.4. Trade-Offs and Synergistic Analyses

Trade-off and synergy analysis can rapidly and qualitatively reveal the correlations between the various ecological service functions and the relationship between stakeholders, and can avoid negative impacts [42]. The Spearman correlation coefficient was used to analyze the correlations between the four ecological service functions. The ArcGIS toolbox was used to create a specified number of random points, and the corresponding service function values extracted from each point were obtained. The resultant data were standardized to eliminate the differential impact of magnitude, then the values for the correlation coefficients and the significance values (*p* values) were obtained through correlation analysis to estimate the trade-off and synergy relationships among the ecosystem services. At the 0.01/0.05 confidence level, when the correlation value of the quantified value of the paired ecosystem service function is greater than 0 it shows a significant synergistic relationship (the two ecosystem service functions change in the same direction), and when it is less than 0 it shows a significant trade-off relationship (the two ecosystem service functions change in the opposite direction). The higher the value, the stronger the correlation of ecosystem service functions.

(12) $$\rho_{s}=\frac{\sum_{i=1}^{N}\left(R_{i}-\bar{R}\right)\left(S_{i}-\bar{S}\right)}{{\left[\sum_{i=1}^{N}{\left(R_{i}-\bar{R}\right)}^{2}\sum_{i=1}^{N}{\left(S_{i}-\bar{S}\right)}^{2}\right]}^{\frac{1}{2}}}=1-\frac{6\sum d_{i}{}^{2}}{N\left(N^{2}-1\right)}$$

(13) $$d_{i}=R_{i}-S_{i}$$

In the expression, *R_i_* and *S_i_*, respectively, represent the corresponding value levels at the observed values of *i*; $\bar{R}$, $\bar{S}$, respectively, denote the average level of variables *X* and *y*; *N* denotes the total number of observations.

## 3. Results

### 3.1. Characteristics of Land Use Change

**Figure 2:**
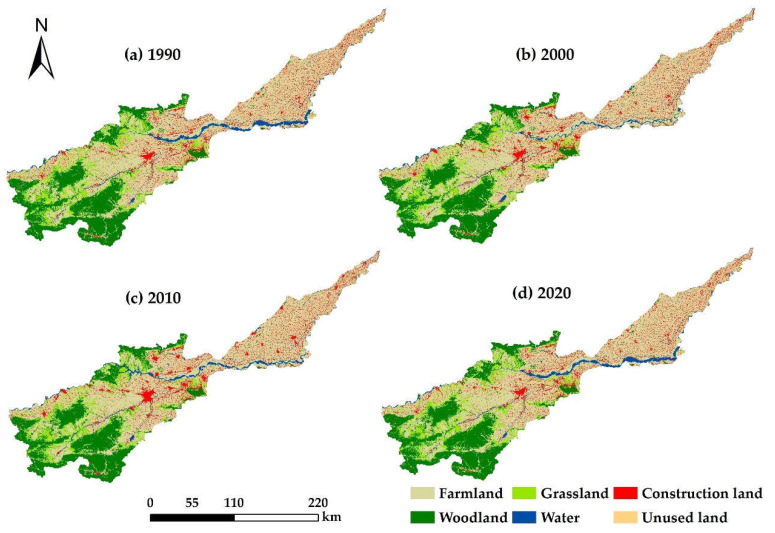
Distribution of land use in the Yellow River Basin (Henan section): (**a**) land use map for 1990; (**b**) land use map for 2000; (**c**) land use map for 2010; (**d**) land use map for 2020.

From 1990 to 2020, the land use types for the Henan section of the basin may be divided mainly into six categories (Figure 2). Among them, farmland was distributed mainly in the plains and the downstream reaches of the basin, which occupied the majority of the area. The higher altitude area in the middle reaches exhibited higher rainfall and lower evapotranspiration, and was most suitable for plant growth. Woodland and grassland were distributed in patches in this area. The areas with water depicted in the figure included the Yellow River and its tributaries, which flowed from west to east. Construction land was distributed mainly in the lower reaches of the river basin and clustered in the middle reaches of the plains area where there was a clear state of agglomeration. Areas of unused land were not evident from inspection of the figure.

The areas of the various land types in the basin from 1990 to 2020 exhibited a decreasing trend over the period, except for construction land, among which farmland and grassland decreased the most (Table 2). The farmland, distributed in the plains and representing 53.68–55.31% of the total land use area, was dominant, and overall the fluctuation in the ratio over the study period was relatively stable. The next highest land use type was woodland, which was concentrated in the higher elevation areas in the middle reaches of the river, with a ratio of 22.74–22.88%, the ratio decreasing marginally from 22.88% in 1990 to 22.74% in 2020. Grassland was clustered and was distributed in the middle reaches of the basin at higher elevations, the ratio for the grassland area ranged from 9.61% to 10.03%, and showed a downward trend from year to year. Woodland and grassland account for approximately one-third of the total land use area. The water bodies were mainly the Yellow River and its tributaries, which decreased from a maximum ratio of 3.86% in 1990 to a minimum ratio of 2.80% in 2000 and then increased year by year. The ratio for the construction land area was 8.61–10.48%, and the overall trend was a gradual increase year by year; construction land was mainly distributed in the middle and lower plain as evidenced by a strengthening of the agglomeration. The ratio of unused land in the study area was 0.04–0.34%, and the ratio decreased over the study period.

**Figure 3:**
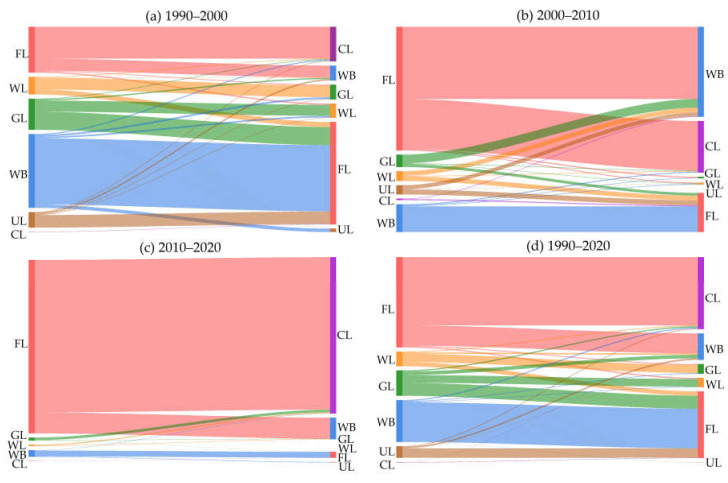
Quantitative change trajectories of land use types in the Yellow River Basin (Henan section): (**a**) trajectory map for 1990–2000; (**b**) trajectory map for 2000–2010; (**c**) trajectory map for 2010–2020; (**d**) trajectory map for 1990–2020. Note: FL, WL, GL, WB, CL and UL represent farmland, woodland, grassland, water, construction land and unused land, respectively. The trajectory lines of different colors represent the flow direction of the corresponding class in a specific period, and the thickness of the lines represents the amount of land conversion. The thicker the lines, the greater the amount of conversion.

The trajectories for the changes in the number of different land use types (Figure 3) indicates that the land use of the Henan section of the basin has changed significantly during the past 30 years. From 1990 to 2000, the conversion of land use types was active. Among them, the transformation of water body was more intense, with a total of 489.10 km^2^ transformed into other land types, of which 89.49% was converted to farmland. There was also a clear outflow trend for grassland, whereas construction land was dominated by an inflow behavior, the total inflow being 229.03 km^2^, of which 89.49% was converted to farmland. The conversion of land use types from 2000 to 2010 was more regular than that of the previous period. The overall outflow of farmland was greater than the inflow as a main feature, and the direction favored water (58.29%) followed by construction land (40.11%). The inflow of construction land was still dominant, mainly from farmland (96.03%). The outflow of woodland, grassland and unused land mainly flowed to farmland, and the outflow of grassland decreased compared with the previous 10 years. From 2010 to 2020, the conversion of land use types was relatively monotonous. A major attribute was the transformation of farmland to other land types, namely construction land (87.09%), water (12.07%), grassland (0.02%) and woodland (0.01%). During the 30 years from 1990 to 2020, there were active transformations among the different land use types and the transformation modes were diverse. The conversion of land use types was frequent, except for unused land, and the inflow and outflow of farmland were relatively intense and occupied a dominant position. Meanwhile, the inflow and outflow of woodland were basically flat, and other land types were dominated by outflow. In the past 30 years, the area of construction land has increased greatly, mainly from farmland (94.47%). It can be concluded that the construction land expanded rapidly due to the overwhelming effect of the increase in population and the associated urban development from 1990 to 2020. Furthermore, the implementation of engineering measures such as returning farmland to forest and grassland achieved useful outcomes. In summary, land use conversion was mainly between construction land and other land types.

### 3.2. Water Yield

#### 3.2.1. Temporal Variation Characteristics of Water Yield

The average annual natural runoff of the Yellow River in the Henan section is 47.4 × 10^8^ m^3^ [43]. After multiple simulations and corrections of the *Z* value, it was determined that when *Z* = 2.6, the simulated water yield was close to the actual situation and the evaluation result was optimal (Table 3). From 1990 to 2020, the total water yields were 42.77 × 10^8^ m^3^, 57.62 × 10^8^ m^3^, 44.47 × 10^8^ m^3^ and 48.54 × 10^8^ m^3^, respectively, which indicate clear fluctuations and an overall increasing trend, the overall increase being 13.49%. The total water yield increased by 1.49 × 10^9^ m^3^ at a rate of 0.03% from 1990 to 2000 and decreased by 1.32 × 10^9^ m^3^ at a rate of 0.03% from 2000 to 2010. The main land use types in the Henan section included farmland, woodland, grassland and construction land. There were significant differences in the land use areas, the size of the evapotranspiration capacity, the water holding capacity of the litter and the water content in the soil, which led to significant differences in water yield capacity. The yield of farmland was the largest and reached the highest in 2000, due mainly to the increase in the area of farmland and rainfall. The water yield for construction land was next and continuously increased over the years, reaching its maximum in 2020, because the construction land area was larger than those in the previous years. The woodland and grassland were larger in area, however, the evapotranspiration was also higher, so the water yield was slightly lower. Water and unused land had the lowest water yield.

#### 3.2.2. Spatial Distribution Characteristics of Water Yield

**Figure 4:**
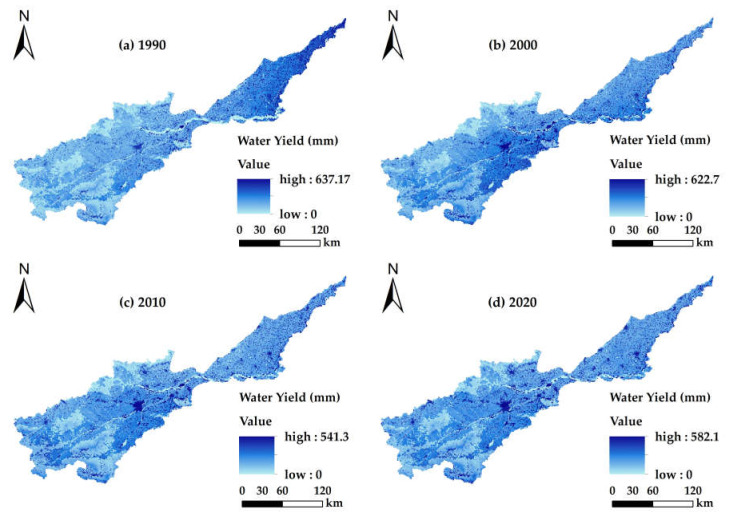
Spatial distribution for the depth of the water yield for the Yellow River Basin (Henan section): (**a**) water yield map for 1990; (**b**) water yield map for 2000; (**c**) water yield map for 2010; (**d**) water yield map for 2020.

Owing to the differences in the annual precipitation, the potential for evapotranspiration and the land use types among the regions, the average water yield for the Yellow River Basin (Henan section) from 1990 to 2020 was generally lower in the southwest and slightly higher in the northeast (Figure 4). Low-value areas were mainly distributed in the southwest of the middle reaches and river systems from west to east of the river basin. The main land use types in these areas were woodland, grassland and water. Although the precipitation was abundant, vegetation transpiration and water evapotranspiration were high, resulting in a low depth for the average water yield. The median area was distributed in the lower-lying plains area in the middle and lower reaches of the basin, and the main land use type was farmland. The rainfall in the middle reaches of this region was low, while the rainfall in the lower reaches was slightly higher, at moderate levels. The transpiration of the aboveground vegetation was lower than those of woodland and grassland, so the average depth of the water yield was moderate. The high-value areas were located mainly in the low-lying plains in the middle and lower reaches of the basin, and gradually clustered over time. The rainfall in this area was moderate and the evapotranspiration was high. There was no vegetation on the construction land to intercept the rainfall, and the evapotranspiration was low. The impermeability of components and materials hindered the infiltration of rainfall to form surface runoff, so the average depth of the water yield was high. The ranking for the average water yield for the land types was as follows: construction land > farmland > grassland > woodland > unused land and water.

### 3.3. Soil Conservation

#### 3.3.1. Temporal Variation Characteristics of Soil Conservation

The soil conservation of the Yellow River Basin (Henan section) in the four periods from 1990 to 2020 were 11.34 × 10^8^ t, 13.73 × 10^8^ t, 12.37 × 10^8^ t and 13.53 × 10^8^ t, respectively (Table 4). Among them, the total soil conservation volume was the highest in 2000 and increased by 2.39 × 10^8^ t with an annual rate of change of 0.02% from 1990 to 2000, from 2000 to 2010 it decreased by 1.36 × 10^8^ t with an annual rate of change of 0.01% and from 2010 to 2020 it increased by 1.16 × 10^8^ t. Over the past 30 years, it has increased by 19.35% with a significant fluctuating trend. The soil conservation in the Yellow River Basin (Henan section) was contributed to mainly by farmland, woodland and grassland, accounting for 96.02–96.87%, mainly because a large proportion of these land types facilitated adsorption due to the presence of aboveground vegetation roots, which effectively minimized soil erosion. The contributions of construction land, water and unused land to soil conservation were low. This was due to both the small area of these land types and there being less vegetation cover, so the ability to prevent silt and soil erosion was low. In 2000, as a result of China’s implementation of soil and water conservation policies and other engineering measures, the soil conservation of farmland, woodland and grassland were at a maximum. With respect to urban development and civil engineering construction, some emphasis was given to the implementation of ecological projects such that the rate of urban greening increased, thus the contribution to soil conservation by construction land gradually increased.

#### 3.3.2. Spatial Distribution Characteristics of Soil Conservation

**Figure 5:**
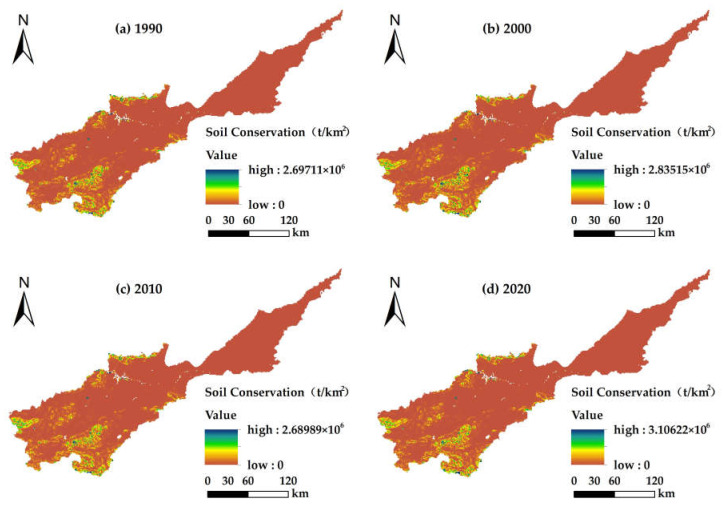
Spatial distribution for the average soil conservation for the Yellow River Basin (Henan section): (**a**) soil conservation map in 1990; (**b**) soil conservation map in 2000; (**c**) soil conservation map in 2010; (**d**) soil conservation map in 2020.

Given the average soil conservation value for the Yellow River Basin (Henan section) (Figure 5), it can be seen that the average soil conservation from 1990 to 2020 showed an upward trend and gradually decreased going from the southwest to northeast. The high value of soil conservation was distributed in the southwestern part of the study area, mainly because the woodland and grassland were at relatively high altitudes. The overground vegetation coverage and human engineering protection measures have enhanced the soil conservation capacity of the area. The median area was distributed mainly in the middle and lower reaches of the plains, where farmland was mostly distributed, and the aboveground grain production could effectively prevent water and soil loss. However, due to factors such as low vegetation coverage and intensified soil erosion caused by human activities, the soil retention capacity of the northeastern part was lower than that of the southwestern part. There was no vegetation cover on water bodies and the soil adsorption was weak, so the east-west section of the Yellow River and its tributaries had a very weak soil conservation capacity and low soil conservation per unit area. The average soil conservation amount in the different land use types was ranked as follows: woodland > grassland > unused land > farmland > water > construction land. Taking 2020 as an example, the average amount of soil conservation in the high-value area was 7.67 × 10^5^–3.11 × 10^6^ t/km^2^, the middle-value area was 1.22 × 10^5^–7.67 × 10^5^ t/km^2^, and the low-value area was not higher than 1.22 × 10^5^ t/km^2^.

### 3.4. Carbon Storage

#### 3.4.1. Temporal Variation Characteristics of Carbon Storage

Based on using the carbon module and ArcGIS, the total carbon storage values for the land use types in the Yellow River Basin (Henan section) from 1990 to 2020 were 1.262 × 10^8^ t, 1.263 × 10^8^ t, 1.252 × 10^8^ t and 1.246 × 10^8^ t, respectively (Table 5). Among them, the total carbon storage amount was highest in 2000, and this increased slightly by 1.10 × 10^5^ t with an annual rate of change of 0.00009% from 1990 to 2000, decreased significantly from 2000 to 2010 by 1.02 × 10^6^ t with an annual rate of change of 0.00081% and from 2010 to 2020 decreased by 6.04 × 10^5^ t with an annual rate of change of 0.00048%. The carbon storage exhibited fluctuating trends, at first increasing and then decreasing, and over the past 30 years the total carbon storage has decreased by 1.20%. Woodland contributed the most to the carbon storage, followed by farmland and grassland, mainly because the vegetation in the woodland and grassland grew luxuriantly and the plant residues were easily accumulated. The vegetation coverage of farmland was lower than that of woodland and grassland, but its area was larger. Water and construction land had the lowest carbon storage.

#### 3.4.2. Spatial Distribution Characteristics of Carbon Storage

**Figure 6:**
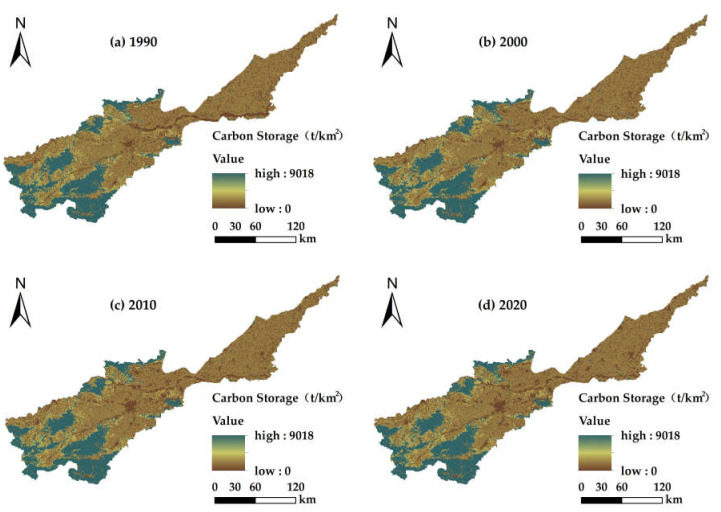
Spatial distribution of average carbon storage in the Yellow River Basin (Henan section): (**a**) carbon storage map for 1990; (**b**) carbon storage map for 2000; (**c**) carbon storage map in 2010. (**d**) carbon storage map for 2020.

From the spatial distribution map of average carbon storage in the Yellow River Basin (Henan section) (Figure 6), it can be seen that the average carbon storage value in the study area has basically not changed from 1990 to 2020. The overall spatial distribution pattern was high in the southwest and low in the northeast, and the low value areas were distributed in the basin and gradually clustered. Because the carbon content of soil and vegetation is determined by the nature of the soil and vegetation itself, it usually does not change dramatically with the environment in the absence of serious external disturbance. The area with high carbon storage values was distributed in the southwest and surrounding areas of the middle reaches of the basin. In this area, woodland and grassland were clustered, vegetation was flourishing and litters were easily accumulated, which made the carbon storage capacity of this region strong, accounting for 69.72–70.26% of the total carbon storage in the study area. The middle area was located in the lower plains of the middle and lower reaches of the Henan section of the basin, where farmland was distributed. The carbon storage due to natural and artificial farming during soil development was second only to woodland, accounting for 29.72–30.24% of the total carbon storage. The low-value area was located in the middle of the basin, and the main land use types included water, construction land and unused land. It has been expanded gradually over the past 30 years and the vegetation coverage on the clustered ground was low, thus, the carbon storage capacity was weak. The carbon storage per unit area of the high-value area was 3395.01–9018 t/km^2^, that of the medium-value area was 990.21–3395.01 t/km^2^, and that of the low-value area was less than 990.21 t/km^2^.

### 3.5. Food Supply

#### 3.5.1. Temporal Variation Characteristics of Food Supply

Based on our calculations, the food supply values for the Yellow River Basin (Henan section) in the four periods from 1990 to 2020 were 0.74 × 10^7^ t, 1.28 × 10^7^ t, 1.88 × 10^7^ t and 2.06 × 10^7^ t, respectively, and in general a gradual increasing trend was observed (Table 6). From 1990 to 2000, the food supply increased by 0.54 × 10^7^ t at a rate of 0.05%, which was a big increase for this period. From 2000 to 2010, the food supply increased by 0.59 × 10^7^ t at a rate of 0.03%. During this period, the areas of farmland and grassland decreased, therefore, the average supply dropped substantially. From 2010 to 2020, the food supply slightly increased by 0.19 × 10^7^ t at a rate of 0.009%. In general, the food supply and the average food supply significantly changed over the period from 1990 to 2020. Over the past 30 years, the food supply increased by 1.32 × 10^7^ t and the average food supply increased by 578.51 t/km^2^ at a rate of 0.03%. In this study, only the food supply of farmland and grassland was calculated. The food supply kept increasing, and the rate of the contribution of the food supply from farmland accounted for more than half the total, indicating that food production from the region’s land increased over time.

#### 3.5.2. Spatial Distribution Characteristics of Food Supply

**Figure 7:**
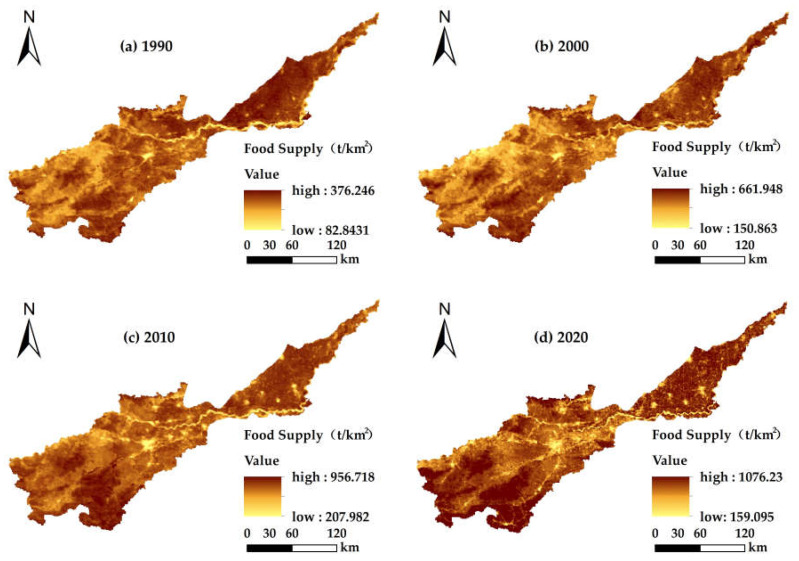
Spatial distribution of average food supply in the Yellow River Basin (Henan Section): (**a**) food supply map for 1990; (**b**) food supply map for 2000; (**c**) food supply map for 2010; (**d**) food supply map for 2020.

With respect to the spatial distribution of the average food supply in the Yellow River Basin (Henan section) from 1990 to 2020 (Figure 7), it was generally high in the eastern and western sides and low in the middle region. The areas corresponding to the low value for the average food supply were located in the middle of the basin, and also distributed erratically (scattered points) in the lower reaches. The main land use types included construction land and water, which had low NDVI values, therefore, the average food supply was very low. However, the minimum food supply gradually increased from 82.8431 to 207.982 t/km^2^ with time, and the distribution of scattered low-value areas gradually expanded into a sheet-like distribution with the expansion of construction land. The high-value areas were located in the lower reaches of the Yellow River and the southwest of the middle reaches. The main land use types were farmland, grassland and woodland with high NDVI values, so the average food supply was high and increased gradually from 376.246 to 1076.23 t/km^2^. Overall, different land use types and food supplies were staggered, which was significant in the lower reaches. With the investment of several basic facilities and technologies such as the construction of high-standard basic farmland and an increase in input efficiencies in the production process—such as the use of organic fertilizers—the land production condition in the region improved, which contributed to a gradual increase in the production potential and food supply of the region.

### 3.6. Multi-Level Ecosystem Service Trade-Offs and Synergies

#### 3.6.1. Trade-Offs and Synergies of Ecosystem Services at the Basin Level

**Figure 8:**
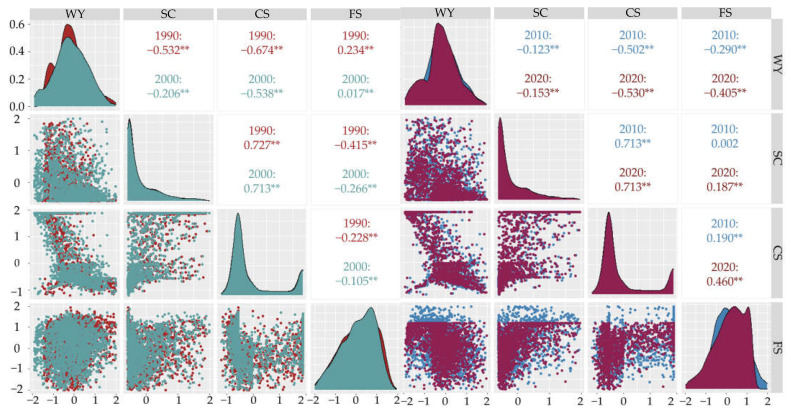
Correlation of ecosystem services in the Yellow River Basin (Henan section). Note: ** at the 0.01 level (two-tailed), the correlation was significant. WY, SC, CS and FS represent the functions of water production, soil conservation, carbon storage and food supply, respectively.

The Spearman rank correlation coefficient matrix showed that the water yield, soil conservation, carbon storage and food supply in the Yellow River Basin (Henan section) from 1990 to 2020 were highly correlated at the confidence level of 0.01, and changed over time. There were six pairs of trade-offs, synergies and neutral relationships among the four ecological service functions in each period. In 1990, there were 4, 2 and 0 pairs, respectively; in 2000, there were 5, 0 and 1 pairs, respectively; in 2010, there were 5, 0 and 1 pairs, respectively; and in 2020, there were 6, 0 and 0 pairs, respectively (Figure 8).

There were significant trade-offs in the water yield—soil conservation and water yield—carbon storage, which gradually increased over time. The relationship of water yield—food supply changed from trade-off to neutral and then became synergistic and gradually strengthened. Soil conservation—carbon storage showed a synergistic relationship with strong correlation. The trade-offs between soil conservation—food supply and carbon storage—food supply weakened into synergistic relationships and then gradually strengthened. As a whole, the trade-off relationships in the Yellow River Basin (Henan section) were dominant in 1990 and 2000, and the synergistic relationships of the ecosystem services in the basin strengthened gradually in 2010 and 2020, among which there was a strong synergy for soil conservation—carbon storage and a stable strong trade-off for water yield—soil conservation. Water yield—food supply, food supply—soil conservation and carbon storage—food supply changed from trade-off to synergy.

#### 3.6.2. Trade-Offs and Synergies of Ecosystem Services at the Landscape Level

**Figure 9:**
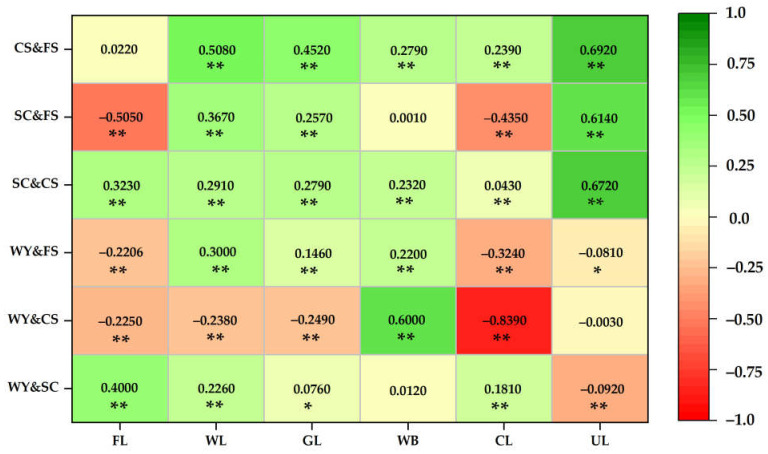
Correlation of ecosystem services among the land use types. Note: (1) ** at the 0.01 level (two-tailed), the correlation is significant; (2) * at the 0.05 level (two-tailed), the correlation is significant; (3) FL, WL, GL, WB, CL and UL represent farmland, woodland, grassland, water, construction land and unused land, respectively; (4) green indicates the synergy between ecosystem services (>0), that is, the ecosystem services in pairs are strengthened or weakened at the same time. Red indicates the trade-off relationship between ecosystem services (<0), that is, one ecosystem service function is strengthened/weakened, and the other ecosystem service function is weakened/strengthened.

The different land use types were distributed in different regions of the Yellow River Basin (Henan section). Water yield, soil conservation, carbon storage and food supply exhibited different correlations for the different land use types, and the ecosystem service functions in the region showed spatial heterogeneity (Figure 9).

Farmland was distributed mainly in the lower reaches of the Yellow River Basin (Henan section) and the middle reaches of the plains. At the 0.01 confidence level, water yield—soil conservation and soil conservation–carbon storage showed significant synergy, while water yield—carbon storage, water yield—food supply and food supply—soil conservation showed significant trade-offs. Woodland and grassland were distributed mainly in the west of the middle reaches, and overall showed a synergistic relationship, in which that of carbon storage—food supply was strong. The water bodies were primarily the Yellow River and its tributaries running from west to east throughout the whole region, and overall synergistic relationship were evident; in particular, the synergy between water yield—carbon storage was strong. The accumulation of construction land was distributed in the middle and lower reaches of the plains, and exhibited a trade-off relationship as a whole. Water yield—carbon storage, water yield—food supply and soil conservation—food supply exhibited trade-off relationships, and the synergy of water yield—carbon storage was extremely strong. Unused land, which was scattered throughout the basin, was small, and soil conservation—carbon storage, soil conservation—food supply and carbon storage—food supply showed strong synergistic relationships.

In general, the ecosystem service functions in the middle and lower reaches of the Yellow River Basin (Henan section) were dominated by trade-offs, while the higher altitude areas of the middle reaches were dominated by synergies. The trade-off of water yield—carbon storage was the strongest for construction land, and the synergy of carbon storage—food supply was strongest for unused land and woodland. In summary, the different land use types were distributed throughout the different regions, and the pairwise service functions showed significant spatial heterogeneities.

## 4. Discussion

### 4.1. Influencing Factors of Ecosystem Service Functions

The frequent human activities and changes in the natural climate lead to changes in the ecosystem services. The continuous growth of population stimulates the growth of potential demand for food and housing that requires the continuous reclamation of land for planting and construction activities, thus further stimulating the growth of land demand. Different land use types have different ecological functions (such as the food supply function of farmland and the soil conservation function of woodland). Human activities cause the mutual transformation of land use types and the change of natural climate such as global temperature and precipitation. Reasonable human activities can promote the healthy development of the ecological environment. However, when the influence of these factors exceeds the bearing capacity of the ecosystem, environmental problems such as ecological degradation will occur to different degrees. The changes in the ecosystem services in the Yellow River Basin (Henan section) were affected by the changes in the land use types, precipitation, temperature and the various human activities [44,45]. According to the above results, the changes in the ecosystem services in this study were closely related to the changes in the land use types. The distribution of farmland in the lower reaches of the basin was relatively dense, which would provide a relatively higher food supply function than other regions. However, due to frequent (rational or irrational) human activities and the evapotranspiration of plant growth and respiration, the soil conservation and water yield capacity were relatively moderate. Woodland and grassland in the higher middle reaches showed a flake concentration distribution due to the effect of the plant roots on the soil, whereby the rainfall and the vegetation litter were readily intercepted. Thus, soil conservation, carbon storage and food supply capacity were relatively high. Moreover, because the consumption of plants was needed to absorb moisture, this was accompanied by a large amount of evapotranspiration, thus, the water yield capacity was very weak. Most of the construction land in the lower plains of the middle and lower reaches of the basin was distributed haphazardly in clumps. The construction materials had strong water impermeability, which effectively prevented rainfall from infiltrating into the ground, hence resulting in the formation of surface runoff. Additionally, because of less vegetation coverage and evapotranspiration on the ground, the water yield capacity in the area was somewhat higher than expected. Changes in the natural climate are also factor which affect ecosystem services. Research has shown that among the factors of influence, such as surface morphology, precipitation, soil erodibility and relevant management policies and measures, the amount of precipitation had a relatively severe effect on soil conservation and water yield [46,47]. The management policies and measures related to human activities had less impact, while the surface morphology and soil erodibility had greater impact on the soil conservation function, among which the rate of coverage of surface vegetation was one of the most important factors [48].

### 4.2. Relationships between Ecosystem Service Functions

Different functions of ecosystem services have significant spatial scale effects, and the interaction between various services might lead to trade-offs and synergistic changes in their relationships [49]. The trade-off for ecosystem services in the Yellow River Basin (Henan section) weakened gradually overall, while the synergy exhibited a strengthening trend. There were mutual interferences and constraints among the different land use types in the different regions, and the different ecosystem service functions exhibited different trade-offs and synergies. Farmland, construction land and unused land had strong trade-offs that accounted for the main position and weak synergies for the ecosystem services, while woodland, grassland and water had strong synergies and weak trade-offs. Due to the nature of farmland, it had strong food supply capacity, but the associated crop growth required relatively high irrigation requirements. In addition, frequent cultivation activities on the land caused the surface to be exposed and reduced the soil conservation capacity of farmland ecosystems. Therefore, there was a significant trade-off between soil conservation and food supply [50]. Furthermore, the increase in the area of farmland and the basic investments required for production and labor improve farm productivity and enhance the food supply function, but this would reduce the carbon storage and vegetation coverage [51]. The overground vegetation in woodland and grassland were abundant and the root systems were well developed, resulting in strong adsorption of soil. Afforestation and planting of new grasslands would not only increase the carbon storage and improve the carbon fixation and oxygen release capacity to help regulate the natural climate, but would also play a significant role in preventing soil erosion (loss of soil and water yield), and could improve the regulation and support of ecosystem services [52,53]. The relationship between ecosystem services was closely related to various functions in different fields, and was caused mainly by the differences between the different land use types and different land use patterns of human beings.

### 4.3. Limitations of Research

Affected by precipitation, evapotranspiration and changes in land use type, the changes observed for the ecosystem services were basically similar, revealing a trend that first increased but then decreased gradually. This study analyzed the characteristics of the spatio-temporal variation of land use and ecosystem services in the Yellow River Basin (Henan section) and the coupling relationships between each service function. In addition, the correlation coefficients for the relationships were visualized with the aid of *R* language. Compared with other ecosystem service research methods, this paper has the following advantages. First, it eliminated previous research based on the Yellow River Basin or administrative boundaries and selected key regions in the Yellow River Basin based on the river basin boundary to carry out ecosystem services research in semi-humid temperate climate zones. Second, we used a longer time series (1990–2020) to analyze its changing rules. Third, it eliminated a single scale and measured the trade-offs and synergies of ecosystem services at watershed and landscape scales, which helped to provide guidance and scientific basis for the management objectives of forest resources on different spatial scales. However, there will be various errors such as engineering measurement factor *P*; clearly, it is difficult to avoid errors based on using results of other researchers. The rainfall erosion factor *R* and the soil erosion factor *K* had errors in the calculation process due to the inaccuracies associated with interpolation. The selection of values for parameters used in the model resulted in errors due to variations in the applicability to the different regions. Furthermore, this study only evaluated water yield, soil conservation, carbon storage and food supply services; other service functions such as habitat quality also play an important role. In future work, it is necessary to address more services to achieve a dynamic and comprehensive evaluation to document and help us explore further the ecosystem service functions of the region. This study only quantified the service material, without quantifying the importance of their functions by value. Comparing the value of service functions with the economic value that they can directly provide can make the results for ecosystem service functions more intuitive and encourage people to be more proactive in protecting the ecosystems. In the future, different scenario simulation schemes may be used to evaluate future change trends, which will provide reasonable scientific basis for regional ecological security protection and sustainable high-quality development of the Yellow River Basin (Henan section).

As an important ecological barrier in China, the Yellow River Basin is the key to maintaining a sustainable social development at regional ecological and economic levels. The Yellow River Basin (Henan section) covers both the middle and lower reaches of the basin, providing various ecological services, including food production, regulating the natural ecological climate and maintaining regional ecological environment security [54]. The management of the basin centers mainly on water resource management, which must be given high priority to ensure a dynamic balance in water quality and quantity. Shortages in water resources will lead to an imbalance of agricultural production and ecological water use in the river basin, and eventually would lead to an imbalance in the related natural hydrological processes. The sustainable and high-quality development of ecology, economy and society are required to build a solid foundation of agriculture, have an abundance of quality water resources, provide green ecological security for forests and grasslands and undertake best practice in safety management including the restoration of mountains and rivers, forests, fields, lakes, grasses and sand. The survey studied the land use change process of the Yellow River Basin (Henan section) from 1990 to 2020, revealing the rule of land use change in 30 years, and analyzed the heterogeneity of ecosystem service functions in different land use types. Based on the above research, policy recommendations are made for the middle and lower reaches of the Yellow River Basin (Henan section) as follows to provide some basic references for regional land use and ecological protection: (1) With reference to the woodland and grassland areas in the middle reaches of the Yellow River Basin (Henan section), which have strong soil conservation and carbon storage capacity, there should be policies in place to return some farmland to forest, to place hillsides off-limits to facilitate afforestation, to plant shelter forests, for restoration of vegetation and for reconstruction to reduce soil erosion caused by surface exposure caused by frequent human activities; there is also a need to strictly prohibit all kinds of deforestation, overgrazing and other destruction of natural vegetation behaviors. (2) The plains area in the middle and lower reaches of the Yellow River Basin (Henan section) have abundant rainfall, low evapotranspiration and strong water yield capacity. It is vital to strengthen the supervision and management requirements of resource development and other construction projects, pay attention to ecological restoration, put an end to various agricultural activities in non-cultivated areas, prohibit overexploitation of groundwater and industrial development that is likely to have harmful effects on water quality and conserve and utilize water resources on a rational basis.(3) Strengthen the protection of farmland resources in the flat plains in the lower reaches of the Yellow River Basin (Henan section), establish a reasonable economic or material compensation mechanism to encourage the protection of farmland, increase the publicity on ecological protection, establish and improve the monitoring and supervision system for ecology at a regional level and improve the management system and decision-making mechanism of ecological protection. According to the different characteristics of land use types and the importance of the ecological service function in the region, a flexible ecological compensation mechanism, which is suitable for the level of economic development, should be formulated in stages. (4) We should attach great importance to the storage and regulation function of water resources in the Yellow River Basin (Henan section), reasonably expand the water storage capacity of the basin, supply water according to demand and adopt a hierarchical management responsibility system to strengthen the unified supply of water resources in order to ensure the reasonable and effective allocation and full utilization of water resources in the basin. (5) Give priority to the planning of land development and ecological environment protection, improve the planning of ecosystem services and ecological environment protection and build a comprehensive adjustment mechanism to balance the relationship between social and economic development and ecosystem services in order for policy makers to give consideration to the improvement of ecosystem services while developing regional economies. According to the above research results, through the ecological environment protection to reduce the harm of natural disasters and promoting the rational and scientific use of natural resources, it is expected to comprehensively curb the deterioration of the ecological environment by 2050, so that the important ecological function areas and species-rich areas in the basin can be effectively protected and restored. Trade-offs between ecological services will be reduced, and synergies will be greatly enhanced, resulting in an overall improvement of the ecological environment. The important ecosystems will be rebuilt and restored, thus realizing a virtuous cycle of natural ecosystems.

## 5. Conclusions

There were significant spatial and temporal diversities in land use and ecosystem services in the Yellow River Basin (Henan section) from 1990 to 2020. Our research revealed the following results: (1) The aggregation of land use structure in the Yellow River basin (Henan section) was significant. Over the past 30 years, the transfer of farmland in the basin has been dominant, with the largest rate of transfer being farmland area while the smallest was unused land. Among them, construction land increased with time, while the other categories were mainly outflow to other land use types. The conversions occurred mainly in grassland, woodland, farmland and construction land. (2) From 1990 to 2020, the water yields in the Yellow River Basin (Henan section) were 42.77 × 10^8^ m^3^, 57.62 × 10^8^ m^3^, 44.47 × 10^8^ m^3^ and 48.54 × 10^8^ m^3^, respectively, which showed a trend that at first increased and then decreased with time; also in the spatial dimension, there was a trend of being low in the southwest and slightly higher in the northeast. The total water yield for the different land use types was as follows: farmland > construction land > woodland > grassland > water and unused land. The total soil conservations were 11.34 × 10^8^ t, 13.73 × 10^8^ t, 12.37 × 10^8^ t and 13.53 × 10^8^ t, respectively. In terms of time, the soil conservation increased at first and then decreased, and in terms of spatial distribution was higher in the southwest at higher elevations, and lower in the northeast plain. The order for the amount of soil conservation in the different land use types was as follows: woodland > farmland > grassland > construction land > water > unused land. The carbon storage for each period were 1.262 × 10^8^ t, 1.263 × 10^8^ t, 1.252 × 10^8^ t and 1.246 × 10^8^ t, respectively, and the spatial distribution pattern did not change significantly. Specifically, the carbon storage of lush vegetation in the southwest was high, that of the plains in the middle and lower reaches was moderate, those for construction land and water were low and showed a trend at first increased and then decreased with time. The total carbon storage in the different land types was in the order: woodland > farmland > grassland > unused land > water. The food supplies were 0.74 × 10^7^ t, 1.28 × 10^7^ t, 1.88 × 10^7^ t and 2.06 × 10^7^ t, respectively. Over time, the food supply increased continuously and the rate of the contribution from farmland became larger. (3) The Spearman rank correlation coefficient showed that the trade-offs for different ecosystem services in the Yellow River Basin (Henan section) were dominant before 2000, and the synergies strengthened gradually after 2000, in which soil conservation—carbon storage showed strong synergy, water yield—soil conservation showed strong trade-off and was relatively stable and water yield—food supply, food supply—soil conservation and carbon storage—food supply changed from trade-off to synergy. (4) Ecosystem service functions have different relationships for the different land use types, and the region presents significant spatial heterogeneity. The ecosystem service functions in the middle reaches and lower reaches of the Yellow River basin (Henan section) were mainly trade-offs, while those in the regions with higher elevations in the middle reaches were mainly synergies. The trade-off between water yield and carbon storage on construction land was the strongest, and the synergy between carbon storage and food supply was the strongest for unused land and woodland.

## Figures and Tables

**Table 1:** Data and sources.

Data	Type	Source	Website Link
Land use type	Grid	Resource and Environment Science and Data Center	https://www.resdc.cn/ (accessed on 20 July 2021)
DEM	Grid	Geospatial Data Cloud	https://www.gscloud.cn/ (accessed on 14 September 2021)
Precipitation, temperature, sunshine, etc.	Num	National Meteorological Science Data Center	http://data.cma.cn/ (accessed on 14 September 2021)
Soil data	Grid	National Qinghai-Tibet Plateau Data Center	http://data.tpdc.ac.cn/zh-hans/data (accessed on 14 September 2021)
Vegetation data	Grid	Resource and Environment Science and Data Center	https://www.resdc.cn/ (accessed on 14 September 2021)
Carbon density data	Num	InVEST Model Manual Guide and References	——

**Table 2:** Area (km^2^) and ratio (%) of land use types in the Yellow River Basin (Henan section) from 1990 to 2020.

Land Use Types	1990		2000		2010		2020	
	Area	Ratio	Area	Ratio	Area	Ratio	Area	Ratio
Farmland	19,898.43	54.29	20,272.34	55.31	19,983.48	54.52	19,672.54	53.68
Woodland	8385.25	22.879	8362.09	22.816	8335.33	22.743	8332.71	22.736
Grassland	3674.53	10.03	3566.16	9.73	3529.15	9.63	3523.52	9.61
Water	1414.17	3.86	1024.58	2.80	1237.48	3.38	1265.40	3.45
Construction land	3154.10	8.61	3380.83	9.22	3552.33	9.69	3842.56	10.48
Unused land	124.14	0.34	44.61	0.12	12.84	0.04	13.88	0.04

**Table 3:** Water yields of land use types for the Yellow River Basin (Henan section) from 1990 to 2020 (10^8^ m^3^).

Land Use Types	1990	2000	2010	2020
Farmland	25.79	33.59	24.77	26.57
Woodland	3.41	6.53	4.80	5.40
Grassland	3.89	5.76	4.75	5.06
Water	0.00	0.00	0.00	0.00
Construction land	9.68	11.74	10.15	11.51
Unused land	0.00	0.00	0.00	0.00
Total	42.77	57.62	44.47	48.54

**Table 4:** Soil conservation of various land use types for the Yellow River Basin (Henan section) from 1990 to 2020 (10^8^ t).

Land Use Types	1990			2000			2010			2020		
	Rkls ^1^	Usle ^2^	Sc ^3^	Rkls	Usle	Sc	Rkls	Usle	Sc	Rkls	Usle	Sc
Farmland	2.50	0.19	2.31	3.07	0.24	2.83	2.69	0.21	2.48	2.84	0.22	2.62
Woodland	7.90	0.46	7.45	9.50	0.55	8.95	8.54	0.49	8.05	9.47	0.54	8.92
Grassland	1.49	0.26	1.23	1.83	0.32	1.51	1.67	0.29	1.38	1.76	0.31	1.45
Water	0.10	0.01	0.09	0.10	0.01	0.10	0.19	0.01	0.18	0.23	0.01	0.22
Construction land	0.26	0.01	0.25	0.34	0.02	0.32	0.29	0.01	0.27	0.32	0.02	0.31
Unused land	0.01	0.00	0.01	0.02	0.00	0.02	0.02	0.00	0.02	0.02	0.00	0.02
Total	12.27	0.93	11.34	14.86	1.14	13.73	13.39	1.02	12.37	14.63	1.10	13.53

Note: ^1^ represents the total amount of possible soil erosion; ^2^ is the real total amount of soil erosion; ^3^ is expressed as total soil conservation.

**Table 5:** Carbon storage (10^7^ t) and average carbon density (t/km^2^) of land use types in the Yellow River Basin (Henan section) from 1990 to 2020.

Land Use Types	1990		2000		2010		2020	
	Average	Total	Average	Total	Average	Total	Average	Total
Farmland	1895.00	3.750	1895.00	3.819	1895.00	3.764	1895.00	3.706
Woodland	9018.00	7.608	9018.00	7.594	9018.00	7.564	9018.00	7.565
Grassland	3403.00	1.245	3403.00	1.209	3403.00	1.195	3403.00	1.191
Water	0.00	0.000	0.00	0.000	0.00	0.000	0.00	0.000
Construction land	0.00	0.000	0.00	0.000	0.00	0.000	0.00	0.000
Unused land	1000.00	0.012	1000.00	0.004	1000.00	0.001	1000.00	0.002

**Table 6:** Food supply (10^7^ t) and average supply (t/km^2^) for the land use types for the Yellow River Basin (Henan section) from 1990 to 2020.

Land Use Types	1990		2000		2010		2020	
	Average	Total	Average	Total	Average	Total	Average	Total
Farmland	1895.00	3.750	1895.00	3.819	1895.00	3.764	1895.00	3.706
Grassland	9018.00	7.608	9018.00	7.594	9018.00	7.564	9018.00	7.565

## Data Availability

The data presented in this study are available on request from the corresponding author.
